# Mechanisms of Cancer Induction by Tobacco-Specific NNK and NNN

**DOI:** 10.3390/cancers6021138

**Published:** 2014-05-14

**Authors:** Jiaping Xue, Suping Yang, Seyha Seng

**Affiliations:** 1 Department of Physiology and Biophysics, University of Illinois at Chicago, Chicago, IL 60612, USA; E-Mail: jxue@uic.edu; 2 Department of Medicine, Beth Israel Deaconess Medical Center, Harvard Medical School, Boston, MA 02215, USA; E-Mail: syang@bidmc.harvard.edu

**Keywords:** tobacco, nitrosamines, cancer

## Abstract

Tobacco use is a major public health problem worldwide. Tobacco-related cancers cause millions of deaths annually. Although several tobacco agents play a role in the development of tumors, the potent effects of 4-(methylnitrosamino)-1-(3-pyridyl)-1-butanone (NNK) and N'-nitrosonornicotine (NNN) are unique. Metabolically activated NNK and NNN induce deleterious mutations in oncogenes and tumor suppression genes by forming DNA adducts, which could be considered as tumor initiation. Meanwhile, the binding of NNK and NNN to the nicotinic acetylcholine receptor promotes tumor growth by enhancing and deregulating cell proliferation, survival, migration, and invasion, thereby creating a microenvironment for tumor growth. These two unique aspects of NNK and NNN synergistically induce cancers in tobacco-exposed individuals. This review will discuss various types of tobacco products and tobacco-related cancers, as well as the molecular mechanisms by which nitrosamines, such as NNK and NNN, induce cancer.

## 1. Introduction

Tobacco use is an epidemic and a global public health problem. One in three cancer-related deaths is attributable to tobacco use in the United States [1]. Approximately 1 billion men and 250 million women are smokers worldwide [2,3]. Tobacco use is declining in most industrialized countries, but overall consumption is increasing with approximately 5.5 trillion cigarettes smoked each year [2], driven in part by substantial widespread use, population growth, and economic development [4]. In the United States, tobacco contributes to the preventable and premature deaths of an estimated 443,000 Americans each year [5]. The World Health Organization estimates, based on the trend of longevity, current smoking trends and increasing adoption of unhealthy lifestyles, that the annual death toll will exceed to 12 million and that there will be 15 million new cancer cases diagnosed annually by 2020 [6].

Tobacco products contain a diverse array of chemicals, including nicotine and carcinogens. The combination of nicotine and these carcinogens is devastating and responsible for millions of preventable and premature deaths worldwide. The impact of tobacco on human health varies depending on the types of tobacco products used and the duration of lifetime exposure. Smokers are exposed to tobacco products primarily by smoking manufactured and/or hand-rolled cigarettes. Non-smokers are exposed to tobacco smoke from the environment, where cigarette smoking occurs. This exposure is known as secondhand, involuntary, passive or environmental exposure. Men and women can be exposed to tobacco by consumption of smokeless tobacco. Nicotine is the principal property of all types of tobacco products and smoke. Nicotine is addictive and non-carcinogenic [7]; however, it is capable of activating various signaling pathways related to tumor promotion [8]. In addition, tobacco products contain carcinogens, including nicotine-derived nitrosamines, which cause cancer or pose risk of cancer in animals and humans [9,10,11,12]. Nitrosamines such as 4-(methylnitrosamino)-1-(3-pyridyl)-1-butanone (NNK) and N'-nitrosonornicotine (NNN), are carcinogenic to humans [13]. NNK and NNN induce carcinogenesis by causing DNA adductions and mutations as well as promoting tumor growth through receptor-mediated effects [3,14]. Exposure and consumption of tobacco products are causally associated with various types of cancers [8,9,10,11,12]. Nicotine and nitrosamines form a devastating and fatal alliance. Nicotine activates the brain’s reward system, eliciting cravings for continued tobacco consumption, and it often is accompanied by carcinogens inducing tumor initiation and progression. Tobacco smoking causes 30% of all cancer mortality in developed countries [15], and smokeless tobacco use is a major cause of cancer in the developing world, particularly southern Asia [13,16]. Exposure to tobacco smoke, secondhand smoke, and/or smokeless tobacco consumption is causally associated with various cancers, for instance, cancers of respiratory, digestive, and urinary systems.

This review will describe types of tobacco smoke and tobacco-related cancers and focus on certain molecular mechanisms by which nitrosamines induce carcinogenesis.

## 2. Tobacco

Tobacco use is epidemic and contributes to preventable morbidity and mortality worldwide. There is convincing evidence that tobacco use is causally associated with various cancers [17]. The following describes the association of different types of tobacco use and cancers.

### 2.1. Tobacco Smoke

Tobacco smoking is the leading cause of cancer-related death in the world, having been associated to approximately 1.2 million deaths annually, and it is linked to 90% of lung cancer cases [18]. Tobacco smoke, derived from combustion of manufactured or hand-rolled cigarettes, contains at least 7000 chemicals [9,10,11,12,19]. While nicotine is generally accepted as non-carcinogenic, it is always accompanied in tobacco by carcinogens [7]. Tobacco smoke contains a number of carcinogens known to cause cancers in animals and humans [18]. These carcinogens are derived from various chemical classes such as polycyclic aromatic hydrocarbons (PAHs), nitrosamines (*i.e*., NNK, NNN), aromatic amines, aldehydes, phenols, volatile hydrocarbons, nitro compounds, and other organic and inorganic compounds [3,18]. Nicotine also promotes cancer by activating signaling pathways facilitating cancer cell growth, angiogenesis, migration, and invasion [8]. Nicotine can undergo chemical conversions into carcinogenic NNK, 4-(methylnitrosamino)-1-(3-pyridyl)-1-butanol (NNAL) and NNN during the process of curing or smoking [7,20]. The majority of nicotine can be metabolized to cotinine by cytochrome P450 (CYP) 2A6, CYP2B6, and aldehyde oxidase [7,20], and the remaining nicotine may be converted to other metabolites such as nicotine-*N*-oxide (NNO) [7,20]. Nicotine and nitrosamines (*i.e*., NNK, NNN,) are implicated in tumor promotion by activating nicotinic acetylcholine receptors (nAChRs) and β-adrenergic receptors (β-AdrRs), leading to downstream activation of parallel signal transduction pathways that facilitate tumor progression [8].

Tobacco smoke is causally linked to lung cancer, laryngeal, oropharyngeal, hypopharyngeal, esophageal, stomach, liver, pancreas, bladder, and ureter cancers and renal pelvis and renal-cell carcinoma, squamous-cell cervical carcinoma, and myeloid leukemia [18,21]. Cigarette smoking also increases the risk of cancers for sinonasal, nasopharyngeal, oral cavity, and colorectal cancers [21]. Cigarette smokers have a higher risk of developing advanced stage and high-grade prostate cancer, which generally indicates a poor prognosis [22].

### 2.2. Secondhand Tobacco Smoke

Secondhand tobacco smoke is also known as environmental tobacco smoke, involuntary smoke and passive smoke. Non-smokers can be exposed to secondhand smoke from different sources, such as in the home, the workplace and outside public buildings. Chemicals similar to those found in direct tobacco smoke have been identified in secondhand tobacco smoke, and out of these, 250 are known to be harmful to human health At least 69 chemicals in secondhand tobacco smoke are carcinogens [12,23,24,25]. Secondhand tobacco smoke also contains nicotine and carcinogens such as nitrosamines (*i.e*., NNN and NNK) [18]. Sidestream smoke (previously uninhaled smoke, e.g., smoke from the burning tip of the cigarette) and mainstream secondhand smoke (smoke that has been inhaled and then exhaled into the environment) are different in their physicochemical properties [18]. The ratios of sidestream to mainstream smoke vary largely depending on the constituents of tobacco products from different manufacturers. For example, nicotine, NNK, and NNN ratios can be 7.1, 0.40, and 0.43, respectively [18]. Secondhand tobacco smoke is causally associated with lung cancer in non-smoking adults [12,24]. In the United States, approximately 3000 lung cancer deaths each year among adult non-smokers are associated with exposure to secondhand smoke [26]. Secondhand tobacco smoke is linked to the increased risk of breast cancer, nasal sinus cavity cancer, and nasopharyngeal cancer in adults and the risk of leukemia, lymphoma, and brain tumors in children [24,26].

### 2.3. Smokeless Tobacco

Smokeless tobacco is unburned tobacco, and is also known as chewing tobacco, oral tobacco, spit or spitting tobacco, dip, and snuff. Users chew or suck the tobacco in their mouth and spit out the juice of the tobacco [27]. Nicotine and other chemical compounds are absorbed through the lining of the mouth. Chemical composition of smokeless tobacco products varies depending on brands and manufacturers. Smokeless tobacco has been considered as a potentially reduced risk substitute for tobacco smoking; however, a study by Hecht *et al*. demonstrates that there is similar exposure to the tobacco-specific carcinogen NNK in smokers and smokeless tobacco users [28]. Smokeless tobacco products contain nicotine [29,30] and carcinogens. At least 28 carcinogens, including nitrosamines (*i.e*., NNK and NNN), are identified in smokeless tobacco products [3,13]. Smokeless tobacco is causally associated with oral, esophageal, and pancreatic cancers [13].

## 3. Tobacco-Specific Carcinogens

### 3.1. Carcinogens

Tobacco products contain a diverse array of chemical carcinogens that cause cancers of various types. To date, more than 60 carcinogens in cigarette smoke have been identified and evaluated by the International Agency for Research on Cancer [31]. Among them, tobacco-specific nitrosamines (such as NNK and NNN), PAHs (such as benzo[*a*]pyrene) and aromatic amines (such as 4-aminobiphenyl) are the prominent carcinogens that have been verified in animal models and positively identified in cigarette smoke [32,33]. Nitrosamines in tobacco products are formed by nitrosation of nicotine and related tobacco alkaloids. The terms NNK for “nicotine-derived nitrosaminoketone” and NNA for “nicotine-derived nitrosaminoaldehyde” were devised to emphasize their relationship to NNN via the common precursor nicotine [34]. Seven tobacco-specific nitrosamines, *i.e*., NNN, NNK, NNAL, N'-nitrosoanabasine (NAT), 1-nitrosoanabasine (NAB), *iso*-NNAL, and 4-(methylnitrosamino)-4-3-pyridyl)butyric acid, have been identified in tobacco products. NNN, NNK, and NAT generally occur in quantities greater than the other compounds. NNN, NNK, and NNAL are evidently the most carcinogenic of these compounds [34,35,36,37,38]. NNK and NNN will be the focus of this review.

### 3.2. Molecular Mechanisms of Nitrosamine-Induced Cancer

#### 3.2.1. NNK and NNN Modulated Tumor Initiation: A Battle between DNA-Adducts Formation and Removal

**Figure 1:**
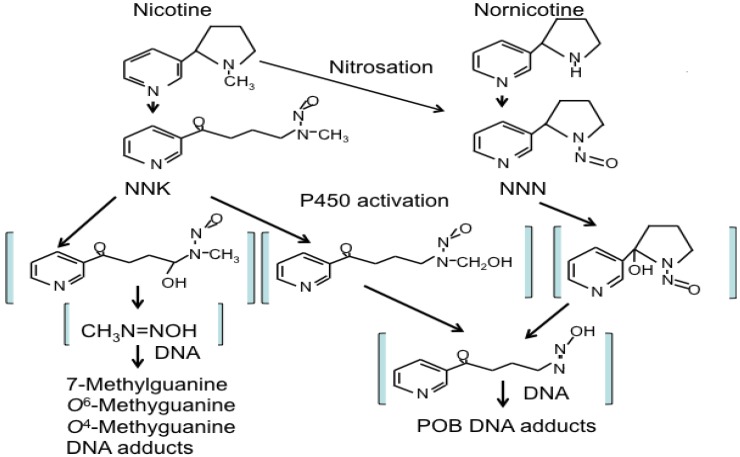
Schematic illustration of the pathways of NNK and NNN metabolism and DNA adduct formation as determined by studies in laboratory animals and humans. NNK: 4-(methylnitrosamino)-1-(3-pyridyl)-1-butanone, NNN: N'-nitrosonornicotine.

Naturally occurring NNK in tobacco smoke is a procarcinogen, an inert form that requires metabolic activation to exert its carcinogenic functions [39,40,41,42]. Multiple CYPs activate NNK to DNA-reactive metabolites that can induce the methylation, pyridyloxobutylation and pyridylhydroxybutylation of nucleobases in DNA and form DNA adducts (Figure 1). α-Methylene hydroxylation of NNK yields methane diazohydroxide [43] and/or the methyldiazonium ion, which reacts with DNA producing mainly 7-*N*-methylguanine (7-mGua) and *O*^6^-methylguanine (O6-mGua) as well as small amounts of *O*^4^-methylthymine. α-Hydroxylation of NNK can occur either at the methyl or methylene carbon. α-Hydroxylation at the methyl carbon produces α-hydroxymethyl NNK, which is stable enough to undergo glucuronidation [44]. It spontaneously loses formaldehyde producing a pyridyloxobutyldiazohydroxide, which reacts with DNA yielding bulky pyridyloxobutylation (POB) adducts [36]. Four of these have been identified. They are 7-[4-(3-pyridyl)-4-oxobut-1-yl]-2'-deoxyguanosine, *O*^2^-[4-(3-pyridyl)-4-oxobut-1-yl]-2'-deoxycytosine, *O*^2^-[4-(3-pyridyl)-4-oxobut-1-yl]-2'-deoxythymidine (*O*^2^-pobdT) and *O*^6^-[4-(3-pyridyl)-4-oxobut-1-yl]-2'-deoxyguanosine (*O*^6^-pobdG) [45]. Figure 1 illustrates schematically NNK and NNN metabolisms and DNA adduct formation.

Reduction of the NNK carbonyl group by carbonyl reductases produces NNAL, which is the predominant NNK metabolite formed *in vitro*. 11-β-Hydroxysteroid dehydrogenase (EC 1.1.1.146), a microsomal enzyme responsible for the interconversion of active 11-hydroxyglucocorticoids to inactive 11-oxo forms, has been identified as one of the carbonyl reductases involved in the reduction of NNK to NNAL. Whether this is the major enzyme responsible for NNK reduction in mammals is not known [46]. NNAL is metabolically transformed in ways similar to that of NNK [34].

Animal studies have shown that NNN specifically causes esophageal and nasal tumors in rats and respiratory tract tumors in mice and hamsters [47,48,49]. Three types of reactions have been observed in NNN metabolism pathways: pyridine N-oxidation, hydroxylation of the pyrrolidine ring (including α-hydroxylation at the 2'- and 5'-positions and β-hydroxylation at the 3'- and 4'-positions) and norcotinine formation [34]. The 2'- and 5'-α-hydroxylation pathways are the major pathways leading to the formation of DNA adducts. 2'-Hydroxy NNN undergoes spontaneous ring opening to produce a pyridyloxobutyldiazohydroxide identical in structure to that formed upon methyl hydroxylation of NNK. 5'-Hydroxylation also yields an electrophilic diazohydroxide, which is expected to react with DNA [39], and the α-hydroxylation reactions of NNN are catalyzed predominantly by CYPs [50].

**Figure 2:**
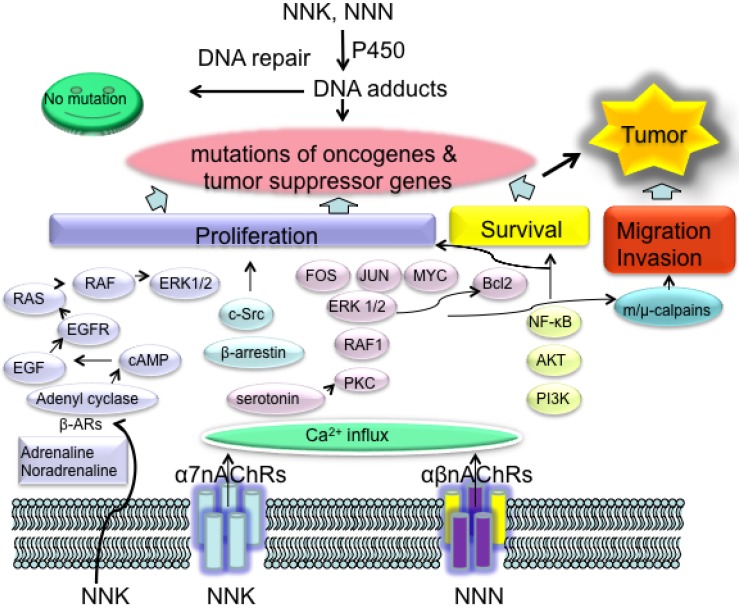
Two essential aspects of NNK- and NNN-induced cancer. Metabolic activated NNK and NNN induce DNA adducts, which can be eliminated by functional DNA repair networks. Unresolved DNA adducts further cause mutations in oncogenes and tumor suppressor genes, which consists of the first step of NNK and NNN specific carcinogenesis. Binding of NNK and NNN to nAChRs promote tumor growth by enhancing and deregulating cell proliferation, cell survival and cell migration as well as cell invasion, which is the second step of NNK- and NNN-induced cancer. The combination of these two aspects of the biological reactions of NNK and NNN provide the condition for tumor development in smokers. NNK: 4-(methylnitrosamino)-1-(3-pyridyl)-1-butanone, NNN: N'-nitrosonornicotine.

Although DNA adduct formation is considered the central step in the process of NNK and NNN carcinogenesis, the capacity of various DNA adducts to induce mutations and chromosomal aberrations varies extensively (Figure 1 and Figure 2). *O*^6^-mGua is a highly pro-mutagenic adduct causing G:C to A:T transitions [51,52]. *O*^6^-mGua adducts can be removed by the DNA repair protein, *O*^6^-alkylguanine DNA-alkyltransferase (AGT; also known as MGMT) or AlkB homologs. AGT overexpression in transgenic mice reduces the formation of K-ras GC→AT mutations and tumors induced by methylating agents [53]. 7-mGua is rapidly removed by base excision repair (BER) as well as by spontaneous depurination. The latter gives rise to apurinic sites that are prone to undergo rapid and error-free repair [54]. In contrast to *O*^6^-mGua, 7-mGua seems to have low mutagenic potency, because there was no correlation between persistence of 7-mGua adduct levels from NNK and incidence of liver tumors in rodents [55]. *O*^6^-pobdG has been shown to be efficiently repaired by AGT both *in vitro* [56] and *in vivo* [57]. If not repaired, *O*^6^-pobdG adducts induce large numbers of G→A and G→T mutations [58].

Although pyridyloxobutyl DNA adducts may also be repaired by nucleotide excision repair (NER) [59] as well as by BER pathways [60], there is no direct evidence for the role of BER in the repair of pyridyloxobutyl DNA damage. However, loss of X-ray repair cross-complementing protein 1 (XRCC1), an important scaffold protein in BER [61], increases the mutagenic and toxic effects of 4-(acetoxymethylnitrosamino)-1-(3-pyridyl)-1-butanone (NNKOAc) [60], which indicates that XRCC1 plays an important role in protecting cells against the harmful effects of these adducts. Similar to BER, loss of xeroderma pigmentosum complementation group A and group C (XPA and XPC), two important components in the NER pathway, reduce the rate of incorporation of [α-^32^P] thymidine 5'-triphosphate into NNKOAc treated plasmid DNA when assayed in NER-deficient cell lysate [62,63]. *O*^2^-pobdT was the only adduct whose removal was affected by the loss of excision repair cross-complementing-2 (ERCC-2 also known as XPD), an essential protein in the NER pathway [64]. Its repair was significantly slower in the absence of ERCC-2 suggesting the importance of NER in the removal of this adduct [60].

Experimental data has suggested that a multistep process of genetic alterations is responsible for NNK- and NNN-induced carcinogenesis. DNA adducts that are misrepaired or not repaired constitute a necessary, although not sufficient, prerequisite for induction of cancer. Initiation and progression of tumorigenesis, however, is complex and involves inactivation of tumor suppressor genes, activation of oncogenes, inflammatory processes as well as alterations in the tissue microenvironment. Fewer than 20% of smokers will get lung cancer. Susceptibility depends in part on the balance between carcinogen metabolic activation and detoxification in the smokers [65]. The genetic polymorphisms in carcinogen-activating genes as well as in DNA repair genes are important determinants of DNA-adduct levels. DNA repair system sets up the second defense line required for eliminating or repairing the lesions of DNA adducts in the genome from the insults of NNK or NNN. An impaired DNA repair system would constitute a significant risk factor for smoking-related cancers. NNKOAc was more cytotoxic in cell lines lacking AGT, BER and NER repair pathways. It also induced more mutations in the hypoxanthine phosphoribosyltransferase gene in BER- and NER-deficient cell lines [60]. Polymorphisms in the DNA repair gene XRCC1 significantly increase the genotoxicity induced by NNK [66]. Polymorphisms in ERCC-2 limit DNA repair efficiency in humans leading to increased frequencies of chromosome aberration in their lymphocytes. Elevations in NNK-induced aberrations were significantly associated with the 312Asn allele. The risk was higher in smokers [67].

Oxidative stress occurs when the productions of oxidant species (mostly reactive oxygen species (ROS) and reactive nitrogen species (RNS) exceed the cellular neutralizing capabilities. The mitochondrial respiratory chain generates the majority of ROS in aerobic cells by incomplete reduction of molecular O_2_ to H_2_O during oxidative phosphorylation, as well as during microsomal and peroxisomal oxidations [68]. In addition, the production of ROS and RNS are also associated with a number of processes such as inflammation, infections and immune reaction [69]. Cigarette smoke contains free radicals such as nitric oxide and mixtures of hydroquinones, semiquinones and quinones, which can induce redox cycling and are present in oxidative damage in smokers [3,65,70]. The mechanisms of NNK- and NNN-induced oxidative stress are not well understood. However, the ability of NNK to induce oxidative stress was evident when increasing levels of 8-hydroxy-2'-deoxyguanosine (8-OHdG) adducts in lung tissues were detected after either oral administration or intraperitoneal injection of NNK into A/J mice and rats [71,72,73]. 8-OHdG is a major pre-mutagenic lesion generated from ROS that is considered a marker of DNA oxidative damage. 8-OHdG is removed by Mmh/Ogg1 gene product, 8-hydroxyguanine DNA glycosylase 1 (OGG1) through the BER pathway [74,75]. Although NNK-mediated ROS induce DNA lesions, another important aspect is ROS-mediated alteration of the microenvironment required for tumor progression. ROS act as signaling intermediates for many normal as well as pathological cellular processes. Constant activation of transcription factors such as nuclear factor kappa-light-chain-enhancer of activated B cells (NF-κB) appears to be one functional role of elevated ROS levels during tumor progression [76].

#### 3.2.2. NNK and NNN Modulated Tumor Promotion and Progression: Creating a Microenvironment for Tumor Growth

nAChRs consists of five subunits with homo- or hetero-pentamers used to form ligand-gated ion channels in the plasma membranes of certain neurons and on the presynaptic and postsynaptic sides of the neuromuscular junction [77]. Nicotine binds to the *α* subunit of nicotinic acetylcholine receptors (nAChRs) as an agonist by mimicking acetylcholine [78]. Nicotine has a higher affinity with α4β2 heteromeric nicotinic acetylcholine receptors (*α*4*β*2nAChRs) than to α7 homomeric nicotinic acetylcholine receptors (*α*7nAChRs) [79]. Accordingly, the biological functions of α7nAChR are increased in smokers, whereas the functions of α4β2nAChR are impaired. Unfortunately, α7nAChR is the most powerful regulator of responses that stimulate cancer cells, whereas the α4β2nAChR regulates predominantly inhibitory actions, resulting in an environment that provides selective support for the development and progression of cancer *in vivo* [80,81]. In addition to nicotine, NNN binds to heteromeric *αβ*nAChRs and NNK to *α*7nAchR, with 5000 times and 1300 times higher affinity than that of nicotine, respectively [82,83]. High levels of the *α*7nAChR expression are found in small cell lung carcinoma (SCLC), as well as in pulmonary neuroendocrine cells (PNECs), whereas heteromeric nAChRs are undetectable [84,85]. In non-small cell lung carcinoma (NSCLC) cells of different histologic subtypes, both hetero- and homomeric nAChRs are found to be expressed at the same time [86].

NNK- and NNN-mediated proliferative potential and anti-apoptotic effect via nAChRs can be alleviated by antagonists α-bungarotoxin (α-BTX) and mecamylamine, respectively. The role of nAChRs in nitrosamine-induced cancer can be further established by competition binding between endogenous ligand and nitrosamine. Recently an endogenous ligand for the *α*7nAChR has been identified as a secreted mammalian Ly-6/urokinase plasminogen activator receptor-related protein (SLURP) 1. In NNK treated cells, the expression levels of SLURP1 and SLURP2 were reduced. Overexpression of SLURP1 or SLURP2 in the cells reduced the nitrosamine-induced colony formation in soft agar while inhibiting the growth of NNK-transformed keratinocytes in mouse xenografts. In competition with NNK and NNN, SLURP1 bound to *α*7nAChR and SLURP2 bound to nAChRs expressing the *α*3 subunit [83,87]. Although all nAChRs are cation channels, they regulate diverse cellular functions in a cell-type-specific manner. This functional diversity of nAChRs is also reflected in cancers of different cellular origins.

Binding of NNK to α7nAChR activated voltage-gated Ca^2+^ channels and caused influx of Ca^2+^ into lung cells, resulting in membrane depolarization [88]. In turn protein kinase C, the serine/threonine kinase RAF1, the mitogen activated kinases extracellular signal-regulated kinase (ERK) 1 and ERK2, as well as the transcription factors FOS, JUN and MYC were activated, which led to the proliferation of PNECs or SCLC (Figure 2). α-BTX, a site-selective antagonist for the α7nAChR, and imipramine, a serotonin reuptake inhibitor, selectively inhibit this signal transduction pathway, indicating that the responses to NNK were facilitated by nicotinic receptor-initiated release of serotonin. Exogenous addition of serotonin activated the same signaling cascade [89,90]. The same signal cascade also accounted for B-cell lymphoma 2 activation leading to NNK inhibited apoptosis in SCLC cells [91].

Although histologic types of lung cancer were significantly associated with cigarette smoking, adenocarcinoma has a stronger association with smoking than other types of lung cancer, such as small cell carcinoma, squamous cell carcinoma and other NSCLC [92]. Consequently, smokers with chronic obstructive pulmonary disease (COPD) are at a particularly high risk of developing SCLC [93]. COPD is an inflammatory lung disease in which expiration of CO_2_ is decreased [94], while α7nAChR levels are upregulated [95]. In COPD, lung α7nAChR is sensitive to a high CO_2_ and low O_2_ environment [96,97,98]. NNK binds preferentially to the sensitized α7nAChR in the COPD lung instead of binding to β-AdrRs in the healthy lung [99,100]. In addition, phosphodiesterase 4, an enzyme that catalyzes the intracellular breakdown of cAMP was found typically overexpressed in the COPD lung [101,102]. The resulting deficiency in intracellular cyclic adenosine monophosphate (cAMP) deprives lung cells of their defense against hyperactive RAF1-mediated signaling. The pulmonary microenvironment in the COPD lung thus selectively favors the development of a neuroendocrine type of lung cancer under positive growth control by α7nAChR [81].

NSCLC cell lines from large-cell carcinoma, squamous-cell carcinoma, and adenocarcinoma, express both hetero- and homomeric nAChRs. Phosphatidylinositol 3-kinase-AKT pathway and NF-κB are activated in response to NNK treatment in NSCLC cell lines, resulting in stimulation of proliferation and inhibition of chemotherapy-induced apoptosis [103,104]. In alveolar type II cell-derived pulmonary adenocarcinomas (PACs), AKT-dependent nicotine-induced resistance to apoptosis was due to the upregulation of survivin (also known as BIRC5) and X-linked inhibitor of apoptosis (XIAP; also known as BIRC4) [105], whereas α7nAChR-mediated stimulation of NSCLC cell proliferation is through activation of β-arrestin–SRC (Figure 2) [106]. In immortalized human bronchial epithelial cells, NNK activates ERK1 and ERK2 signal transduction pathway, signal transducer and activator of transcription 1(STAT1), NF-κB, and GATA binding protein 3 (GATA3), whereas NNN activates only GATA3 and STAT1 [107].

In addition, NNK is also an agonist of β-AdrRs and directly binds to them with high affinity. In the absence of penicillin and streptomycin that have been shown to interfere with β-AdrR signaling, NNK stimulated growth, and migration of small airway epithelial cells; the PACs derived from them are stimulated through β-AdrR-initiated cAMP signaling that transactivates the epidermal growth factor receptor (EGFR) (Figure 2) and cooperates with non-genomic estrogen receptor-β signaling [99,108,109]. A nAChR-mediated systemic increase in adrenaline and noradrenaline, which are β-adrenergic agonists, may additionally stimulate the development of this type of PAC. Administration of adrenaline in hamsters significantly promoted NNK-induced small airway-derived PAC, which was a strong argument for this hypothesis [81,100].

The inhibitory neurotransmitter γ-aminobutyric acid (GABA) inhibits β-AdrR-initiated cAMP signaling cascade at the level of adenylyl cyclase and effectively blocked DNA synthesis and cell migration. In turn, the release of GABA is regulated by α4β2nAChR, which is desensitized in smokers [110,111] and additionally downregulated in PACs by NNK, leading to a deficit in GABA [112]. The desensitization of this receptor is enhanced by estrogens and phyto-oestrogens [113]. The predominance of PAC in women and NNK-induced tumors in OGG knockout female mice may therefore, at least in part, be the result of estrogen impaired α4β2nAChR function. Moreover, NNK induced cell migration and invasion occurred in both SCLC and NSCLC through ERK1–ERK2-dependent phosphorylation of m-calpains and μ-calpains [114].

Genotoxicity and tumor promotion environment are two essential conditions for tobacco specific nitrosamines-induced cancer. Recently it was shown that the simultaneous expression of oncogenic K-ras, p53 knockdown, and mutant EGFRs were insufficient to confer a full malignant phenotype in bronchial epithelial cells [115]. NNK induces nearly identical numbers of mutation and comparable levels of mutagenic DNA adducts in both susceptible and resistant lungs suggesting a pro-tumor environment is essential for tumor progression. The upregulation of nAChRs and concomitant desensitization of α4β2nAChR in smokers shifts the balance in favor of α7nAChR signaling with strong direct and indirect stimulatory effects on cancer cells, whereas the release of GABA, which counteracts many of these effects, is reduced. This universal switch from balanced neurotransmission to cancer-stimulating neurotransmission is unstoppable once it occurs; blocking one signaling pathway or even removing the primary cancer will not stop the runaway α7nAChR train [81].

## 4. Conclusions

Causal association between tobacco use and cancers is well established. Tobacco smoke contains 7000 chemicals, and of which at least 60 are carcinogens. The human health risk caused by tobacco smoking is not limited to smokers, but also to non-smokers who are exposed to environmental tobacco smoke, causing cancers in adults and increasing cancers in children. Similar levels of carcinogenic NNK exposure are found in tobacco smokers and smokeless tobacco users, therefore the smokeless tobacco is harmful and may not be a reduced risk substitute for tobacco smoking.

Most constituents of tobacco smoke, detoxified or neutralized by metabolizing enzymes, are converted to more water-soluble products, which can be excreted from the human body. However, during this process, certain reactive compounds may be formed as intermediates which may covalently bind to nucleophilic sites in DNA, causing DNA adducts. The DNA adducts can evade the repair system, and can cause miscoding during DNA replication resulting in a permanent mutation in the DNA sequence. The mutation can occur in a cellular oncogene or in a tumor suppressor gene, altering the normal growth control mechanisms, which may lead to uncontrolled proliferation, further mutations and cancer.

Exposure to any type of tobacco is associated with, and/or increases the risk of, various cancers. Tobacco smoke contains a mixture of nicotine, carcinogens and toxicants. Nicotine is not a direct chemical carcinogen, however, it causes addiction leading to the chronic exposure to tobacco smoke that increases cancer risk for tobacco users. While carcinogens, such as nitrosamines, induce cancer by causing gene mutations and/or DNA and protein adducts, nicotine promotes cancer progression by activating signaling pathways that facilitate cancer cell growth, angiogenesis, migration, and invasion. The nicotine and carcinogen alliance is detrimental to human health, costs billions in direct medical care, causes loss of productivity, and is responsible for millions of preventable and premature deaths each year.

A better understanding of the distinct mechanisms by which tobacco induces carcinogenesis may potentiate the discovery of new biomarkers, enhance the development of sensitive methods to identify trace amounts of tobacco-specific carcinogens, facilitate effective epidemiologic studies, and help guide the evolution of public health and health care policy toward implementing improved approaches for the prevention of tobacco-related cancers.

## Abbreviations

NNK: 4-(methylnitrosoamino)-1-(3-pyridyl)-1-butanone
NNN: N'-nitrosonornicotine
NNAL: 4-(methylnitrosoamino)-1-(3-pyridyl)-1-butan-1-ol
PAH: polycyclic aromatic hydrocarbons
POB: pyridyloxobutyl
PHB: pyridylhydroxybutyl
O6mGua: *O*^6^-methylguanine
O4-mTh: *O*^4^-methylthymine
7-mGua: 7-*N*-methylguanine
AGT: *O*^6^-alkylguanine DNA-alkyltransferase
